# PROTECTIVE EFFECT OF LIFETIME EXPERIENCES ON FUNCTIONAL STATUS IN YOUNG-ONSET DEMENTIA

**DOI:** 10.1093/geroni/igz038.436

**Published:** 2019-11-08

**Authors:** Tram N Pham, Lauren Massimo, Katheryn A Cousins

**Affiliations:** University of Pennsylvania, Philadelphia, Pennsylvania, United States

## Abstract

Patients with Frontotemporal degeneration (FTD), a common form of young-onset dementia, experience decline in cognitive, social and daily functioning as the disease progresses. Research shows that lifestyle factors may be an important modifiable risk factor for dementia, but this has not been well studied in FTD. In this study, we test the hypothesis that lifetime experiences, including education, occupation, and leisure activities, are associated with better functional status in individuals with FTD. We also evaluated the relationship between timing of experiences (early, mid-life, and late-life) and functional status. Thirty-five patients (mean age 61.6±8.7; 74% male; mean disease duration 3.4 ± 2.6; mean MMSE 24.0 ± 5.5) completed the Lifetime of Experiences Questionnaire (LEQ), a comprehensive assessment of lifelong cognitive lifestyle, and the Clinical Dementia Rating Scale (CDR), which was used to assess functional status. Linear regression tested the relationship between cognitive lifestyle and functional status, with age and disease duration included as covariates. Higher total LEQ score was associated with better functional status (lower score on CDR) (β = -0.047, p = 0.009). While Young Adulthood LEQ score was not significantly associated with total CDR (β = -0.047, p = 0.176), both Mid-life (β = -0.117, p = 0.011) and Late-life (β = -0.133, p = 0.013) LEQ score significantly contributed to functional status. Our results indicate that functional status is mediated in part by cognitive lifestyle and that experiences accumulated in mid-life and late-life have a greater effect on functional status at time of diagnosis.

